# Anatomical Workspace Study of Endonasal Endoscopic Transsphenoidal Approach

**DOI:** 10.1515/med-2019-0060

**Published:** 2019-10-19

**Authors:** Sorayouth Chumnanvej, Duangkamol Pattamarakha, Thanwa Sudsang, Jackrit Suthakorn

**Affiliations:** 1 Center for Biomedical and Robotics Technology (BART LAB), Department of Biomedical Engineering, Faculty of Engineering, Mahidol University, Salaya, Thailand; 2 Neurosurgery Division, Department of Surgery, Faculty of Medicine Ramathibodi Hospital, Mahidol University, Bangkok, Thailand; 3 Department of Diagnostic and Therapeutic Radiology, Faculty of Medicine Ramathibodi Hospital, Mahidol University, Bangkok, Thailand

**Keywords:** CT imaging, endonasal endoscopic transsphenoidal, Robotic workspace, Skull base, Sphenoid sinus

## Abstract

**Purpose:**

To determine the workspace through an anatomical dimensional study of the skull base to further facilitate the design of the robot for endonasal endoscopic transsphenoidal (EET) surgery.

**Methods:**

There were 120 cases having a paranasal sinus CT scan in the database. The internal volumes of the nasal cavities (NC), the volumes of the sphenoid sinuses (SS), and the distance between the anterior nasal spine and base of the sellar (d-ANS-BS) were measured.

**Results:**

The Pearson correlation coefficient (PCC) between the relevant distances and the volumes of the right NC was 0.32; between the relevant distances and the volumes of the left NC was 0.43; and between the relevant distances and volumes of NC was 0.41; with a statistically significant difference (p < 0.001). All PCCs had a statistically significant meaningful difference (p < 0.05).

**Conclusion:**

The volume of NCs were significantly correlated with distances (p < 0.05). The safest and shortest distance to guide the robotic arm length in the EET approach could be represented by d-ANS-BS. This result was also used as primary information for further robotic design.

## 1 Introduction

The endonasal endoscopic transsphenoidal (EET) approach is now globally accepted [1, 2, 3] and is indicated for lesions in the sellar region [6, 7, 8, 9]. Because of the development of an endoscope with optimal illumination and magnification, this approach provides potential advantages for removing pituitary tumors and lesions near the sellar region or anterior cranial fossa [10, 11, 12, 13, 14]. Compared with the transcranial approach, EET is a minimally invasive and less traumatic approach that provides excellent visualization [15, 21, 22, 23]. Moreover, in an era of minimally invasive surgery, a robotic-assisted procedure used with the EET approach has greater accuracy, precision, and safety [25, 26, 27, 28]. Skull base surgery is one of the fields of greatest interest in robotic-assisted surgery [29, 30, 32]; previous studies have shown the effectiveness and feasibility of its application in this procedure [23, 25, 26, 27, 28].

This innovative EET approach is performed with robotic manipulators that are controlled by a telesurgical system [30, 31]. Furthermore, patient safety is of paramount importance so neurosurgeons have to be well trained to prevent surgical complications [18]. Removing lesions in the sellar region, particularly with a robotic-assisted surgery system, requires a thorough understanding of the intranasal and skull base anatomy because of the extremely deep and narrow surgical anatomical path in this procedure [26, 30]. The primary objective of this study was to design an anatomical dimensional study of the volume and distance around the skull base area for a robotic system for use with the EET approach.

An anatomical configuration study is very much needed. Current knowledge regarding the anatomical structure relevant to the EET approach is mainly based on postmortem or imaging studies [1, 10]. Among imaging techniques, computerized tomography (CT) has the potential to examine the basal skull and intranasal structures in several aspects, particularly for preoperative evaluation of the bony parts [33, 34]. Additionally, CT imaging provides greater accuracy and safety for studying skull base anatomy to identify and classify the EET approach workspace [35, 36, 37].

Study of the intranasal and skull base anatomy surrounding the sellar region by CT imaging was also a primary objective. Our objectives also included measuring the trajectory-to-target distance and classifying the workspace of the EET approach. To establish and support the design concepts of a surgical robot, an anatomical dimensional study– particularly concerning the volumes of the nasal cavities and the distances involved in the intraoperative field of the EET approach–was conducted. The correlations between the volumes and distances involved were essential. Determining the safest and shortest trajectory-to-target distance and classifying the surgical robotic workspace in the EET approach for further clinical application was the secondary objective of the study.

## 2 Methods

This study was conducted after receiving institutional review board approval and in conjunction with the Department of Biomedical Engineering, Faculty of Engineering, Mahidol University. Data were collected from CT scan files and medical records. Based on a retrospective review from January 1, 2011 through March 31, 2013, 518 cases were identified where patients who had undergone CT scans of the orbit using a 64-slice MDCT system (SOMATOM Sensation 64; Siemens, Forchheim, Germany) and a 320-slice MDCT (Aquilion ONE; Toshiba, Tokyo, Japan) at Ramathibodi Hospital, Mahidol University. Of these, 384 patients were excluded because of abnormalities or tumors that had invaded the skull base structure or having had previous surgery. Table 1 provides the inclusion and exclusion criteria. From the 134 patients after exclusions, 120 patients whose consecutive CT scans of the orbit fulfilled the inclusion criteria were analyzed. As area, volume, and the distance are clearly defined in the ORBIT CT, it has been used for the direct visualization and the determination of the bony structure and sellar region[38].

**Table 1:** Inclusion and exclusion criteria

SI. no	Inclusion criteria	Exclusion criteria
1	Male or female	Patients who had undergone previous sinonasal surgery
2	Age ≥ 18 years who had undergone CT scan of the orbit with slice thickness 1 mm with coronal and sagittal reformation.	Patients who had obstructive lesions, pathology or fracture of facial bones, palate, nasal cavity or sellar region.
3	No known history of intranasal and/or sellar pathology	Problems in transferring data from CT scan of the orbit to the Volume Viewer Package on Advantage Workstation 4.4.
4	All scans were reviewed and confirmed to have normal intranasal structures and sellar anatomy	

**Figure 1:**
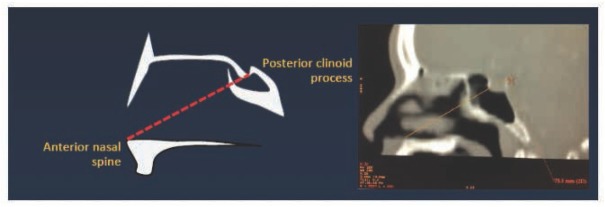
Measurement of the linear distance between the anterior nasal spine and the posterior clinoid process on Advantage Workstation 4.4.

The first step involved image acquisition of the CT scans of orbits. The images were analyzed at 80–120 kV in the axial plane with 1-mm slice thickness and with coronal and sagittal reformation. The data collected included the volumes of the nasal cavities and sphenoid sinuses, and the distances between the anterior nasal spine and the posterior clinoid (Figure 1). The anatomical landmark is the anterior nasal spine and the base of sellar at posterior clinoid process. A line was drawn between the anterior nasal spine and the posterior clinoid process; the distance of the line was determined. Measurements were performed on a median sagittal image in which the nasal septum is visible.

**Figure 2:**
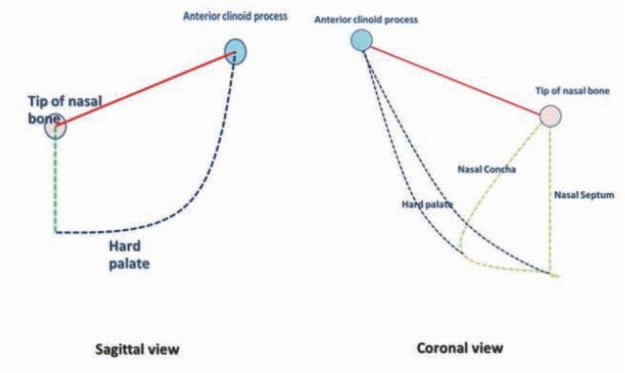
Diagram of nasal cavity alignment.

**Figure 3:**
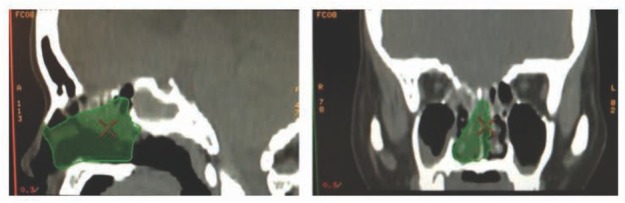
Images relevant to the linear distance between the anterior nasal spine and the anterior clinoid process for the ETT approach.

The sequential CT data sets were measured using the Volume Viewer Package on the Advantage Workstation 4.4 (GE Healthcare, Little Chalfont, UK). Using this software, the segmented volumes of the nasal cavities and the sphenoid sinuses, and the distances between the anterior nasal spine and the posterior clinoid were calculated, as shown in (Figure 2 and 3).

The initial nasal alignment is the imaginary reference line linking the tip of nasal bone and anterior clinoid process, which can be considered as the superior boundary. The posterior boundary can be represented by a second line, which was developed by identifying an imaginary point on the sagittal plane at which the base of sphenoid joins the hard palate. The anatomical landmarks of the anterior and inferior boundary are the anterior nasal spine and the hard palate, respectively. The medial and lateral boundaries are the nasal septum and nasal concha, respectively.

In the second step, the locations and terminology for imaging analysis were defined. “Distance” was defined as the linear distance between the anterior nasal spine and the posterior clinoid. “Volume” was defined as the volume of the nasal cavities or the sphenoid sinuses. All CT scans of the orbit and measurements were interpreted by a neuroradiologist and a neurosurgeon.

All variables were analyzed using descriptive statistics, including means and standard deviations (SD) for analysis of the volume and distance data. The linear relationships between distances and volumes were determined using Pearson’s product-moment correlation coefficient. These coefficients and descriptive statistics were computed using Stata Statistical Software: Release 12 (StataCorp, College Station, TX, USA). After data analysis was completed, the trajectory-to-target and workspace were determined for clinical application.

**Ethical approval**: Cadaveric-based experiments were conducted and an Ethical Approval statement is enclosed as electronic supplementary material.

## 3 Results

One hundred twenty cases (60 males and 60 females) were analyzed. The volumes of the nasal cavities and the sphenoid sinuses, and the distances between the anterior nasal spine and the base of the sellar region were determined. As shown in Table 2, the total mean volumes of the right and left nasal cavities were 25.47 ± 4.31 cm^3^ and 26.20 ± 4.21 cm^3^, respectively. The total mean volume of the sphenoid sinuses was 13.48 ± 4.78 cm^3^. The total mean

**Table 2:** Mean volumes of the right and left nasal cavities and sphenoid sinuses relative to the mean distances between the anterior nasal spine (ANS) and sellar region by sex.

Sex	Mean volume of right nasal cavity (cm^3^)	Mean volume of left nasal cavity (cm^3^)	Mean volume of sphenoid sinus (cm^3^)	Mean distance between ANS and sellar region (mm)
Male (n = 60)	26.89 ± 4.89	27.20 ± 4.51	14.95 ± 4.88	81.67 ± 4.52
Female (n = 60)	24.05 ± 3.06	25.19 ± 3.66	12.00 ± 4.23	78.21 ± 3.67
Total (n = 120)	25.47 ± 4.31	26.20 ± 4.21	13.48 ± 4.78	79.94 ± 4.52

distance between the nasal spine and the base of the sellar region was 79.94 ± 4.52 mm.

In the male patient group, the mean volumes of the right and left nasal cavities were 26.89 ± 4.89 cm^3^ and 27.20 ± 4.51 cm^3^, respectively. The mean volume of the sphenoid sinuses was 14.95 ± 4.88 cm^3^. The mean distance between the anterior nasal spine and the base of the sellar region was 81.67 ± 4.52 mm. For the female patients, the mean volumes of the right and left nasal cavities were 24.05 ± 3.06 cm^3^ and 25.19 ± 3.66 cm^3^, respectively. The mean volume of the sphenoid sinuses was 12.00 ± 4.23 cm^3^. The mean distance between the anterior nasal spine and the base of the sellar region was 78.21 ± 3.67 mm. There was a statistically significant difference (p < 0.001) between the mean distance in males and females, but no clinical difference [39]. The Pearson correlation coefficients between the distances and volumes are shown in Table 3.

**Table 3:** Pearson correlation coefficients for distances and volumes (n = 120).

Variable	r	p
Distances between the anterior nasal spine and sellar region and volumes of the right nasal cavity.	0.43	<0.001
Distances between the anterior nasal spine and sellar region and volumes of the left nasal cavity.	0.32	<0.001
Distances between the anterior nasal spine and sellar region and volumes of the sphenoid sinus	0.25	<0.05

p < 0.05 for significance.

The correlation coefficient was 0.43 for the distances between the anterior nasal spine and base of the sellar and the volumes of the right nasal cavities, 0.32 for the distances between the anterior nasal spine and base of the sellar and of the volumes of the left nasal cavities, and 0.41 for the distances between the anterior nasal spine and base of the sellar and volumes of both right and left nasal cavities. Taking safety factors into consideration, the point at the base of sella is taken to be in the midline throughout the study. All of the correlation coefficients were statistically significant (p < 0.001).

## 4 Discussion

**Figure 4:**
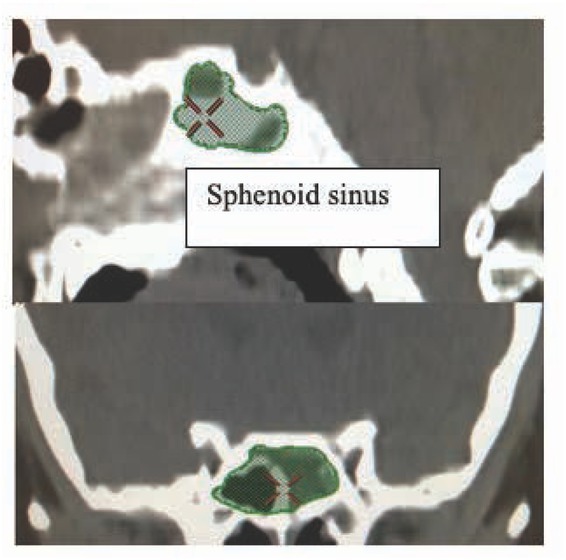
Images relevant to the volume measurement of the sphenoid sinus on Advantage Workstation 4.4.

**Figure 5:**
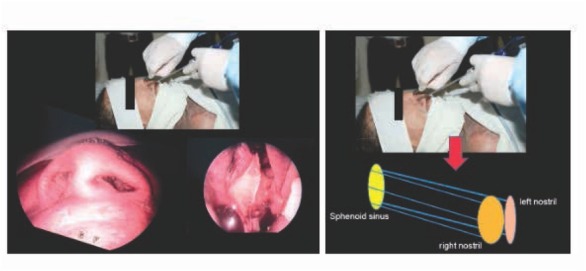
Images and a cylindrical diagram which are relevant to the EET approach.

**Figure 6:**
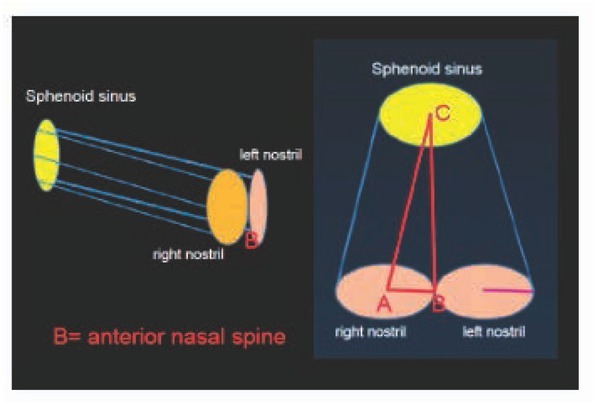
Cylindrical diagrams relevant to the EET approach showing the significant reference lines.

Because the Pearson correlation coefficients were positive, the volumes were assumed to be a constraint on the robotic workspace for the EET approach [15,16,21] (Figure 4). Based on the study objectives, an uncomplicated algorithm was used. First, a diagram was created from the EET approach. A cylindrical diagram was developed using the right and left nasal cavity volumes. The distances between the anterior nasal spine and the posterior clinoid (BC), (Figures 5 and 6) are known. The volumes of the right and left nasal cavities can be analytically estimated through the product of the circular areas shown and the BC (Figure 6).

**Figure 7:**
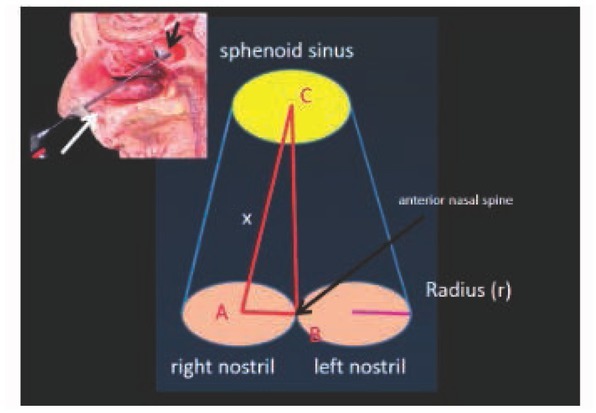
Image and a cylindrical diagram with reference lines relevant to the algorithm.

The radius (r) of the nostrils can be determined. Using the Pythagorean theorem (Figures 6 and 7), the distance AC, or x, was calculated. Therefore, BC and AC were estimated to be almost identical, as shown in Table 4.

**Table 4:** Statistics pertaining to the estimated distances between BC, AC and radius.

Nasal cavity volume (cm^3^)	Distance between ant.nasal spine and clinoid(BC) (cm)	Radius (r) (cm^3^)	AC distance (x) (cm)	Error (cm)
Average group right and left				
29.78	7.54	1.12	7.62	0.083
21.16	8.45	0.89	8.49	0.047
30.41	7.54	1.13	7.63	0.085
21.99	8.45	0.91	8.49	0.049
Male group right and left				
31.74	7.72	1.14	7.80	0.084
21.96	8.62	0.90	8.67	0.047
31.71	7.72	1.14	7.80	0.084
22.69	8.62	0.92	8.67	0.048
Female group right and left				
27.11	7.45	1.08	7.53	0.077
20.99	8.19	0.90	8.24	0.050
28.85	7.45	1.11	7.54	0.082
21.53	8.19	0.92	8.24	0.050

Note: refer to Fig. 7

The cylindrical model is based on the concept that the safest and shortest distance to guide the robotic arm length in the EET approach could be represented by the distance between the anterior nasal spine and the sphenoid sinus. Using the correlation between the distances and volumes as in the cylindrical model (Figures 5 and 6), the target and trajectory were known and were of particular concern for robotic pathway design[23,29,31,32]. In addition, there are some limitations involving this technique related to surgical instrumentations and limited work space [35, 36, 37]. The workspace boundary could be described along with the surgical approach in addition to the volume constraint [37,40,41]. Satisfactory information about the workspace characteristics makes it a practical new modality for robotic workspace classification.

The authors postulated a practical reason to define the volume constraint on the robotic workspace. As a result, the EET workspace was identified and classified during this research including: (1) the available workspace (AWS), the workspace which is defined by the limitation of clinical anatomy or volume constraint, ie, the cylindrical model (Figure 5); and (2) the trajectory-to-target workspace (TWS), the workspace which is defined by the entry point to the target based upon the clinical anatomy and the surgical approach, ie, the cone-shaped model (Figure 6 and 7); and (3) the universal workspace (UWS), the workspace defined based upon the area of interest, ie, the area for robot installation or the area that might be identical to the AWS. In addition, the TWS should be smaller than the AWS because of the target point at the end of the workspace. In conclusion, this information and the clinical needs that should be met to achieve its acceptance are also considered to be fundamental information for designing the robotic workspace and as the initial information for robotic design [42].

## 5 Conclusions

Regarding the EET approach, the volumes of the nasal cavities were significantly correlated with distances (p < 0.05). However, there were no clinically significant differences between the distances in males and females. The study reports the workspace determination, pertaining to the designing of robot for EET approach. Other outcomes regarding the study included the safest and shortest distance to guide the conceptual design of robotic arm in the EET approach, which could be represented by the distance between the anterior nasal spine and the sphenoid sinus.
